# The exosome controls alternative splicing by mediating the gene expression and assembly of the spliceosome complex

**DOI:** 10.1038/srep13403

**Published:** 2015-08-26

**Authors:** Lin Zhang, Yufeng Wan, Guobin Huang, Dongni Wang, Xinyang Yu, Guocun Huang, Jinhu Guo

**Affiliations:** 1 State Key Laboratory of Biocontrol, Key Laboratory of Gene Engineering of the Ministry of Education, School of Life Sciences, Sun Yat-sen University, Guangzhou 510006, China; 2 Centre for Circadian Clocks, Medical College, Soochow University, Suzhou 215123, China

## Abstract

The exosome is a complex with exoribonuclease activity that regulates RNA surveillance and turnover. The exosome also plays a role in regulating the degradation of precursor mRNAs to maintain the expression of splicing variants. In *Neurospora*, the silencing of *rrp44*, which encodes the catalytic subunit of the exosome, changed the expression of a set of spliceosomal snRNA, snRNP genes and SR protein related genes. The knockdown of *rrp44* also affected the assembly of the spliceosome. RNA-seq analysis revealed a global change in bulk splicing events. Exosome-mediated splicing may regulate alternative splicing of NCU05290, NCU07421 and the circadian clock gene *frequency* (*frq*). The knockdown of *rrp44* led to an increased ratio of splicing variants without intron 6 (I-6) and shorter protein isoform small FRQ (s-FRQ) as a consequence. These findings suggest that the exosome controls splicing events by regulating the degradation of precursor mRNAs and the gene expression, assembly and function of the spliceosome.

Most eukaryotic genes contain coding exons and non-coding introns. During mRNA biogenesis, the introns are removed from the precursor mRNA (pre-mRNA), and the exons are joined in a process called splicing. Splicing is mediated by the spliceosome complex, a large RNA-protein complex. The spliceosome is comprised of U1, U2, U5, and U4/U6 small nuclear ribonucleoprotein particles (snRNPs) and an additional group of non-snRNP proteins^1^. The budding yeast spliceosome contains approximately 100 different proteins, and the spliceosome complex in mammals contains over 300 different snRNP proteins and spliceosome-associated proteins. The structure and function of the spliceosome are under dynamic control^1^,^2^.

The splicing of an exon can be either constitutive or alternative. In constitutive splicing, the exon is always included in the mature mRNA; while in alternative splicing (AS), an exon is not always included in the mRNA. AS occurs in multicellular organisms and has contributed to the evolution of highly complex proteomes by diversifying the gene expression products^3^. Alternative splicing is essential for many important physiological processes in eukaryotes and is often regulated in tissue- or developmental stage-specific manners.

The regulation of mRNA degradation is crucial for the temporal control and fidelity of gene expression. The exosome complex, which consists of ten subunits, is a 3′ → 5′ exoribonuclease machinery that controls the turnover of all classes of RNAs^4^,^5^,^6^. The structures and functions of the exosome are highly conserved in eukaryotes. In budding yeast, the exosome core itself lacks RNase activity; instead, RNA degradation is achieved by Rrp44/Dis3 or Rrp6; these two proteins exert 3′ → 5′ exoribonuclease activity. Rrp44 is a processive hydrolytic exoribonuclease related to *E. coli* RNase R, a member of the RNase II (RNase B) family of hydrolytic exonucleases^7^,^8^. Rrp6 is a distributive hydrolytic exoribonuclease that mainly regulates the decay of nuclear RNAs^9^. In fungi and invertebrates, the genome only contains one copy each of the *rrp6* and *rrp44* genes. However, plants and vertebrates contain multiple copies of analogues of these genes^6^. The exosome has little RNase activity in the absence of cofactors, which may prevent the cell from inappropriate RNA degradation^10^. As a cofactor, the TRAMP (Trf4/5–Air1/2–Mtr4 polyadenylation) complex recruits the exosome to the RNA substrates and stimulates the exonucleolytic activity of the exosome^11^.

Most organisms possess circadian clocks that synchronize daily rhythmicities in metabolism, physiology, and behavior^12^. In recent decades, a growing body of evidence has suggested that alternative splicing plays a critical role in the regulation of circadian clocks in multiple species^13^,^14^,^15^,^16^,^17^,^18^. In the circadian clock of *Neurospora crassa*, WHITE COLLAR 1 (WC-1) and WC-2 are two positive components that form the WHITE COLLAR COMPLEX (WCC) to activate the transcription of the negative component FREQUENCY (FRQ) encoded by the *frq* gene^19^,^20^. The exclusion or inclusion of I-6 of the *frq* pre-mRNA leads to the production of small FRQ (s-FRQ) or large FRQ (l-FRQ) isoforms, respectively^21^,^22^. The ratio of s-FRQ to l-FRQ negatively correlates with the ambient temperature and is crucial for the fine-tuning of circadian periodicity and temperature compensation of the circadian clock^22^,^23^,^24^. The exosome is also implicated in the regulation of the circadian clock. *Neurospora* RRP44 directly regulates the turnover of *frq* mRNA and might account for the prolonged circadian period in the *rrp44* knockdown strain^25^. In *Neurospora*, FRQ-interacting RNA helicase (FRH) is the homolog of yeast Mtr4, which plays multiple roles in regulating the circadian clock at both the post-transcriptional and post-translational levels^25^,^26^,^27^,^28^,^29^.

During splicing, the exosome also regulates the degradation of pre-mRNAs. In yeast, the poorly spliced pre-mRNAs as well as the RNAs that contain longer introns are more susceptible to degradation^30^,^31^. The yeast exosome has been previously shown to regulate the processing and maturation of snRNAs^5^,^32^, suggesting that the exosome might be involved in the regulation of splicing. However, whether and how the exosome regulates alternative splicing remains elusive.

In this work, we showed that the exosome regulates the gene expression of spliceosomal components and assembly of the spliceosome. Furthermore, in addition to the known role during pre-mRNA elimination, the exosome mediates splicing via the spliceosome complex. This work also sheds light on the role of the exosome in the regulation of pre-mRNA splicing and the circadian clock.

## Results

### Exosome mediates expression of splicing factors

*Rrp44* encodes the catalytic subunit of the exosome complex which regulates gene expression at the post-transcriptional level. To identify genes controlled by the exosome genome-wide, we conducted transcriptomic RNA sequencing (RNA-seq) in the ds*rrp44* strain, in which the expression of *rrp44* is silenced by the expression of a double-stranded RNA complementary to the *rrp44* upon the addition of 0.01M QA^25^. The same strain without QA treatment was also subjected to RNA-seq as a control. Upon *rrp44* knockdown, 2521 genes were up-regulated and 1180 genes were down-regulated. To identify the biological processes regulated by the exosome, we conducted bioinformatic analysis of the genes differentially expressed with or without QA treatment by searching the DAVID database^33^. KEGG pathway analysis indicated that the knockdown of *rrp44* led to the modification of a variety of biological functions, including pyrimidine metabolism, RNA polymerase, and spliceosome (Table 1), suggesting that the expression of spliceosomal genes might be controlled by the exosome.

**Figure 1:**
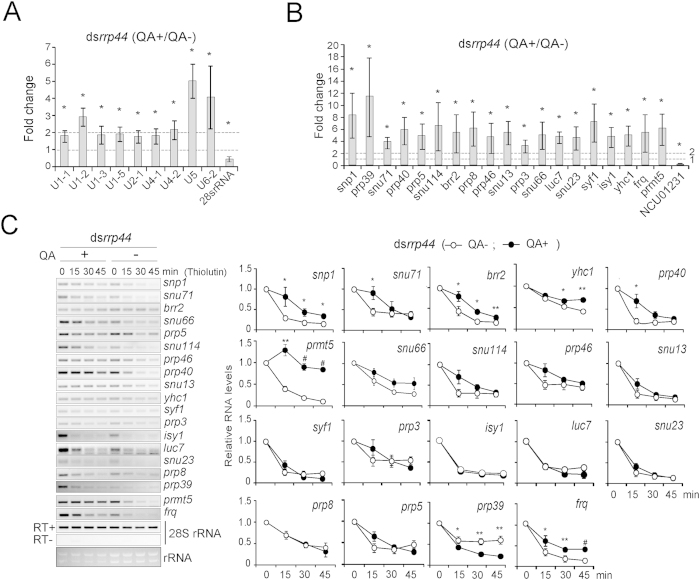
The expression of certain spliceosomal components and spliceosome-associated genes is up-regulated in ds*rrp44* strain. (**A**,**B**) qRT-PCR analyses of the expression of snRNA (**A**) and snRNP (**B**) genes in the ds*rrp44* strain, QA+ versus QA−. (**C**) RT-PCR of 18 mRNAs encoding spliceosomal components in the ds*rrp44* strain in the presence and absence of QA. Thiolutin was added to block the transcription. Samples were harvested at 0, 15, 30, and 45 min after thiolutin addition. Left panel: Representative results of three independent experiments. Right panel: Densitometric analysis of the mRNA decay results, the RNA levels at 0 min was set to 1. Data represent means ± SD (**A**,**B**) or SEM (**C**) of three independent experiments, *p < 0.05, **p < 0.01, ^#^*p* < 0.001, QA + versus QA−.

The spliceosome contains numerous small nuclear ribonucleoproteins (snRNPs) and small nuclear RNAs (snRNAs)^3^,^34^. The RNA-seq analysis showed that the levels of 16 mRNAs (*snp1*, *prp39*, *snu71*, *prp40*, *prp5*, *snu114*, *brr2*, *prp8*, *prp46*, *snu13*, *prp3*, *snu66*, *luc7*, *snu23*, *isy1* and *syf1*) that encode the components of the spliceosome complex and *protein arginine methyl transferase* (*prmt5*) mRNA that encodes a spliceosome-associated protein were significantly increased in the ds*rrp44* strain (Fig. 1B; Table 2). PRMT5 is a type II protein arginine methyltransferase that regulates splicing by transferring methyl groups to arginine residues in spliceosomal Sm proteins^16^. The expression of *yhc1* was slightly but not significantly increased, and we also included this gene in the verification experiments. An increase in the expression of each of these genes was observed by qRT-PCR (Fig. 1B), which confirmed the RNA-seq results. However, the values of fold change largely differ from the qRT-PCR results, and this deviation can be explained by the fact that the RNA-seq results were obtained from only one pooled sample. The *frq* RNA levels were also elevated in the *rrp44* knockdown strain using qRT-PCR analysis, consistent with a previous report^25^. The level of NCU01231 mRNA decreased in the presence of QA, which served as another control. We also evaluated the expression of snRNA genes (*U1-1*, *U1-2*, *U1-3*, *U2-1*, *U4-1*, *U4-2*, *U4-5*, *U5* and *U6-2*), and the qRT-PCR results demonstrated that the expression levels of all tested snRNA genes were significantly elevated in ds*rrp44* strain (Fig. 1A). These data imply that the expression of these snRNA genes is under the control of RRP44, which is consistent with the data obtained from yeast^32^.

To identify the spliceosomal genes that are directly degraded by the exosome, we investigated the mRNA turnover of these 18 genes in the ds*rrp44* strain with or without the addition of QA. To analyze mRNA decay, the transcription inhibitor thiolutin was added to block the transcription^25^. The silencing of *rrp44* led to a marked decrease in the degradation rates of *snp1, snu71*, *brr2*, *prp40*, *yhc1*, and *prmt5* mRNAs, which suggested that the transcripts of these genes are direct targets of exosome-directed degradation. As expected, *frq* mRNA displayed a reduced turnover when *rrp44* expression was repressed, which agrees with previously reported results^25^. In contrast, the stabilities of *snu114*, *prp46*, *snu66, prp5, snu13, syf1, prp3, isy1, snu23, luc7*, or *prp8* mRNA did not significantly change (Fig. 1C). Unexpectedly, the degradation rate of *prp39* increased upon *rrp44* knockdown (Fig. 1C), which indicated that the mRNA decay of these genes is not under the direct control of RRP44. These data reflect that mRNA decay is redundantly and competitively regulated by different complexes, e.g., the 3′→5′ and 5′→3′ machineries. Thus, the exosome both directly and indirectly acts on the expression of a set of spliceosomal genes.

### Spliceosomal components regulate alternative splicing

*Neurospora* contains 1.7 introns per gene, and the average intron size is 134 nucleotides; both of these values are much lower than those in higher eukaryotes^35^. To verify the roles of *Neurospora* spliceosomal components in the regulation of splicing, we generated knockdown strains that expressed lower levels of *prp5* (ds*prp5*), *prmt5* (ds*prmt5*), and the snRNA gene *U4-2* (ds*U4-2*). PRP5 is an RNA-dependent ATPase that regulates pre-spliceosome formation and the release of spliced mRNA from the spliceosome^36^. *U4-2* is one member of the *Neurospora* U4 snRNA gene family; U4 snRNA pairs with U6 snRNA in the U4/U6·U5 tri-snRNP complex^1^.

**Figure 2:**
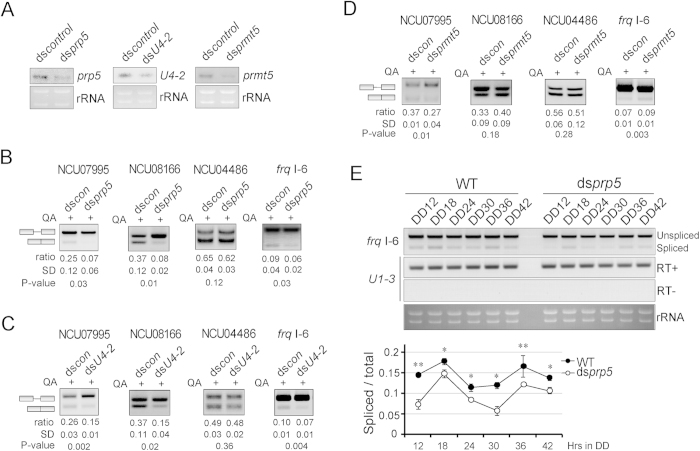
Splicing of representative genes in the knockdown strains ds*prp5*, ds*U4-2* and ds*prmt5*. (**A**) Northern blot validation of the knockdown strains of ds*prp5*, ds*U4-2*, and ds*prmt5*. (**B**–**D**) RT-PCR results showing the splicing of NCU07995, NCU08166, NCU04486 and *frq* I-6 in the strains ds*prp5* (**B**), ds*U4-2* (**C**), and ds*prmt5* (**D**). Ratios of spliced/total are shown. (**E**) RT-PCR analysis of the alternative splicing of *frq* I-6 at DD12 to DD42 in increments of 6 h in WT and ds*prp5* strains. Data represent means ± SD of three independent experiments.

The addition of QA induced the silencing of the targeted gene in these knockdown strains^26^. The down-regulation of *prp5*, *U4-2*, and *prmt5* expression in the knockdown strains upon QA addition was validated by northern blot analyses (Fig. 2A). To analyze the effect of the knockdown of these genes on splicing, the levels of the spliced and unspliced species of the three selected genes, NCU07995, NCU08166 and *frq*, were analyzed by RT-PCR. The *frq* gene comprises eight splicing variants in total, and those variants with or without I-6 can be translated into l-FRQ or s-FRQ proteins, respectively. We selected these genes because the RNA-seq results revealed that the alternative splicing of these genes was differentially regulated in the ds*rrp44* knockdown strain. As a control, the splicing of NCU04486, which was not altered in the ds*rrp44* strain, was also evaluated. The level of mature NCU07995 mRNA, but not NCU04486 mRNA, was decreased in the ds*prp5*, ds*U4-2*, and ds*prmt5* strains in the presence of QA, while the mature NCU08166 mRNA level was also reduced in ds*prp5* and ds*U4-2*. The spliced variants of *frq* without I-6 was decreased in ds*U4-2* and ds*prp5* strains and increased in ds*prmt5* (Fig. 2B–D). The levels of *frq* transcripts oscillate in constant dark (DD)^20^. We further measured the splicing of *frq* I-6 from DD12 to DD42 in increments of 6 h in WT and ds*prp5* strains, and the results showed that the ratio of *frq* variants without I-6 oscillated and the level of variants without I-6 in ds*prp5* was significantly lower than that in WT (Fig. 2E).

Arginine/serine-rich non-snRNP splicing factors (SR proteins) contribute to the regulation of splicing specificity^37^. The RNA-seq data also indicate an increase in the levels of several SR protein genes or SR protein related genes (data not shown). We measured the expression of four SR protein genes (*srsf4*, *sr140*, *rbm39* and *srsf15*), and in agreement, the results showed induction of levels of all the four genes. The results from mRNA degradation showed a significant decrease in the mRNA degradation of *srsf4* and *sr140*, suggesting they are direct targets of exosome. By contrast, the stabilities of *rbm39* and *srsf15* showed no significant change (Supplemental Fig. 1). Taken together, these data suggest that the spliceosome plays an important role in the regulation of alternative splicing in *Neurospora*.

### Exosome mediates the assembly of spliceosome complex

**Figure 3:**
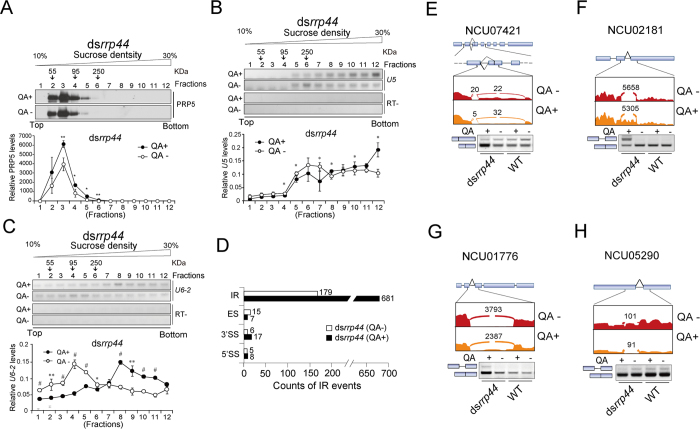
RRP44 regulates the assembly of the spliceosome complex and alternative splicing. (A) PRP5 protein was detected by western blot in the fractions from sucrose gradients of the ds*rrp44* strain grown in the presence or absence of QA. Upper panel: Representative PRP5 profiles revealed by sucrose fractionation and western blot analysis. Locations of the protein markers (55KD, 95KD, 250KD) from parallel gradients are indicated above the panel. Bottom panel: Densitometric quantification of PRP5 distribution. (**B**,**C**) Distribution of *U5* (**B**) and *U6-2* (**C**) snRNAs in the ds*rrp44* strain grown in the presence or absence of QA. Upper panels: Representative results of three independent experiments. Bottom panels: Quantification of three independent experiments. The total value from 12 densitometric analyses of each experiment was normalized to be 1.0. (**D**) Differentially regulated AS events in ds*rrp44* strain grown in the presence or absence of QA as derived from RNA-seq analysis. Events are classified into groups based on the modes of alternative splicing, and samples that have higher amounts of the longer of the two isoforms caused by alternative splicing were considered to be up-regulated. (**E**–**H**) RT-PCR results showing the differential splicing in four representative genes in ds*rrp44* strain grown in the presence or absence of QA. RT-PCR results from the WT strain grown in the presence or absence of QA are shown as controls. Up panels: Sashimi plots of the four AS genes in ds*rrp44* with or without QA. Sashimi plots showing RNA-seq reads mapping to the loci of indicated genes. Heights of the bars represent overall read coverage. Splice junctions supported by ≥10 split reads are displayed as loops. Bottom panels: Representative results of three independent RT-PCR results. Data represent means ± SD of three independent experiments. *p < 0.05, **p < 0.01, ^#^*p* < 0.001.

The induction of spliceosomal genes observed in the ds*rrp44* strain treated with QA suggests that the exosome might regulate the assembly and function of the spliceosome. To address this possibility, we performed sucrose density gradient sedimentation to fractionate the total protein extracts from ds*rrp44* grown in the presence or absence of QA. The distribution of PRP5 protein in the sucrose density gradient fractions was evaluated by western blot analysis, and the results showed that the PRP5 levels increased in the fractionated samples from organisms grown in QA, suggesting that the knockdown of *rrp44* not only affects the levels of spliceosomal mRNA but also the protein level of this spliceosomal component. In addition, the distribution of PRP5 in fractions number 3–6 was significantly higher in response to QA treatment compared to untreated samples (Fig. 3A).

We then investigated the distribution patterns of snRNA *U5* and *U6-2* in samples fractionated by sucrose sedimentation. We extracted the total RNAs from the fractions and conducted RT-PCR to examine the *U5* and *U6-2* snRNA levels in the sucrose sedimentation samples. The RT-PCR results demonstrate the differential distribution patterns of *U5* and *U6-2* snRNAs in ds*rrp44* grown in the presence or absence of QA; the levels of both snRNAs increased in higher-density fractions upon QA treatment (Fig. 3B,C). Because snRNAs exert their functions in complex with snRNP proteins^38^, this change in the distribution of an snRNA upon the silencing of *rrp44* supports our hypothesis that the spliceosome assembly is altered in RRP44-deficient organisms.

### Knockdown of *rrp44* leads to extensive changes in AS events

To further address our hypothesis that the exosome regulates splicing, we compared the alternative splicing (AS) events in the ds*rrp44* strain grown in the presence or absence of QA derived from RNA-seq. The results showed that the knockdown of *rrp44* led to changes in at least four types of AS events: intron retention (IR), exon skipping (ES), alternative 5′ splice site selection (A5′SS), and alternative 3′ splice site selection (A3′SS). Changes in IR events predominated among the total 918 changed splicing events, although differences in ES and A5′/3′SS were detected (Fig. 3C; supplemental Table 1). As in other eukaryotes, such as the fungus *Fusarium graminearum* and the plant *Arabidopsis thaliana*^39^,^40^, the most common AS events in *Neurospora* appear to be IR.

The analysis revealed that 179 IR events decreased while 681 IR events increased in the ratio of unspliced transcripts in the ds*rrp44* strain treated with QA compared to the strain without QA treatment (Fig. 3C, supplemental Table 1). Because the exosome is implicated in pre-mRNA degradation^30^,^31^, some of the 681 IR events might result from the stabilization of pre-mRNAs due to *rrp44* knockdown, while some of the 179 IR events might result from changes in alternative splicing. These observations demonstrate that the exosome contributes to bulk splicing events.

We conducted RT-PCR to validate the RNA-seq results. Four representative genes were evaluated, including NCU07421, NCU02181, NCU01776 and NCU05290. Of these genes, NCU07421 encodes an RNA-binding and nuclear transport protein, NCU02181 and NCU01776 encode ribosomal protein components, and NCU05290 encodes an orotate phosphoribosyltransferase. After *rrp44* knockdown, NCU07421 showed a change in the selection of an alternative 3′ splice site (Fig. 3E). NCU02181 and NCU01776 showed higher levels of intron retention (Fig. 3F,G), while NCU05290 showed an increase in splicing (Fig. 3H), in agreement with the RNA-seq data.

**Figure 4:**
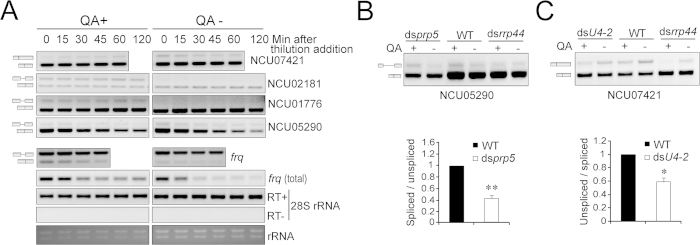
Regulation of AS by exosome and spliceosomal components. (**A**) RT-PCR results showing the changes in splicing variants of tested genes after thiolutin addition in the ds*rrp44* strain in the presence or absence of QA. Samples were harvested 0, 15, 30, 45, 60 and 120 min after thiolutin addition. (**B**) Upper panel: RT-PCR results showing NCU05290 splicing in ds*prp5*, WT and ds*rrp44* in the presence or absence of QA. Bottom panel: Densitometric quantification of ratios of spliced/unspliced species of NCU05290 in ds*prp5* and WT. (**C**) Upper panel: RT-PCR results showing NCU07421 splicing in ds*U4-2*, WT and ds*rrp44* in the presence or absence of QA. Bottom panel: Densitometric quantification of ratios of unspliced/spliced species of NCU07421 in ds*U4-2* and WT. Data represent means ± SD of three independent experiments. *p < 0.05, **p < 0.01.

To further investigate the degradation of the pre-mRNAs and spliced variants of the genes of interest, we treated the samples with thiolutin and examined the levels of pre-mRNA and spliced variants at a series of time points after thiolutin addition. The unspliced mRNAs of NU02181 and NCU01776 were almost undetectable in the absence of QA, but their expression was elevated in the presence of QA (Fig. 4A). Because the exosome is known to be responsible for eliminating pre-mRNAs^30^,^31^, these data suggest that these unspliced species might represent the pre-mRNAs of the tested genes, and their rapid degradation is controlled by RRP44, which requires less than 15 min to complete. Instead, the degradation of both the unspliced and spliced species of NCU05290 is likely controlled by RRP44 (Fig. 4A). As such, the decrease in its unspliced species might be attributed to the altered splicing upon *rrp44* knockdown. Regarding NCU05290, the knockdown of *prp5* decreased the ratio of the spliced species, and the increased level of *prp5* in the ds*rrp44* strain might consequently at least partially account for the increase in the spliced species (Fig. 4B).

Regarding NCU07421, which bears A3′SS, both the levels of the unspliced and spliced species showed no significant degradation upon *rrp44* inhibition (Fig. 4A), suggesting that the change in its splicing ratio is not due to the degradation of pre-mRNA. Though QA treatment also induced a decrease in the unspliced tanscripts of NCU07421 in the WT strain for unknown reason, the ratio of the unspliced tanscripts of NCU07421 was significantly lower in ds*U4-2* (Fig. 4C). These data suggest that the ratio of the NCU07421 splicing variants might be controlled by exosome-mediated splicing via the spliceosome. Despite the induction of *U4-2* (Fig. 1A), the level of the unspliced variant of NCU07421 was markedly decreased in ds*rrp44* (Fig. 4C), which might be attributed to a disturbance of snRNA maturation due to exosome inhibition^5^,^32^. Taken together, these data, as exemplified by NCU05290 and NCU07421, suggest that in addition to its role in regulating the pre-mRNA degradation, the exosome may control alternative splicing by mediating the gene expression, assembly and function of the spliceosome.

### Regulation of alternative splicing of circadian clock gene *frq*

*frq* transcripts with and without I-6 encode l-FRQ and s-FRQ, respectively, and both l-FRQ and s-FRQ are critical for the circadian clock in *Neurospora*^21^,^22^,^23^,^24^,^41^. The degradation of total *frq* RNA is controlled by the exosome (Fig. 4A)^25^. In addition to the increase in the level of total *frq* transcripts, the ratio of splicing variants with I-6 was significantly decreased (Fig. 4A), which agrees with the RNA-seq data (Bayes factor = 1E^12^, supplemental Table 1) and suggests that the exosome may regulate *frq* splicing in addition to its degradation.

**Figure 5:**
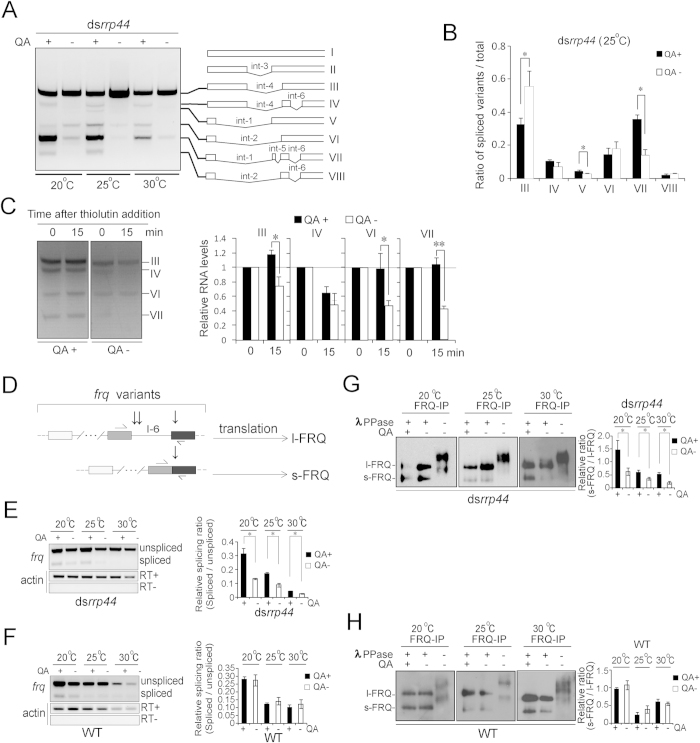
RRP44 differentially controls the degradation and splicing of *frq* variants. (**A**) RT-PCR showing the splicing profiles of the ds*rrp44* strain at three temperatures in the presence or absence of QA. Except for variants I and II, which were almost undetectable, variants III-VIII are labeled. (**B**) Quantification of the ratios of spliced variants versus total transcripts of the results from panel A (25 °C). (**C**) RT-PCR results showing the degradation of *frq* variants (left) and densitometric quantification of variants III, IV, VI and VII at 0 and 15 min (right). The RNA levels at 0 min were set to be 1. (**D**) Schematic representation of *frq* I-6 splicing and the corresponding protein products l-FRQ and s-FRQ. Horizontal arrows denote the relative location of PCR primers flanking *frq* I-6. Vertical arrows denote the locations of the three initiation codons. (**E**,**F**) RT-PCR results and densitometric quantification of *frq* I-6 splicing in ds*rrp44* (**E**) and WT (**F**) strains grown in the presence or absence of QA. (**G**,**H**) Comparison of s-FRQ*/*l-FRQ ratios in ds*rrp44* (**G**) and WT (**H**) strains grown in the presence and absence of QA. FRQ was immunoprecipitated and treated with λ PPase prior to analysis. The samples in the absence of QA without λ PPase treatment served as loading controls. Data represent means ± SD of three independent experiments. *p < 0.05, **p < 0.01.

The *frq* gene comprises eight splicing variants in total, and variants I, II, III, V and VI, but not others, contain I-6^23^. As such, the decrease in the level of *frq* variants that contain I-6 upon *rrp44* knockdown reflects the influence of the exosome on the mixture of different variants. We conducted RT-PCR with primers that spanned all the introns (1–6) to analyze the influence of the exosome on individual splicing variants in ds*rrp44*, and the results showed that the profiles of splicing variants consistently and dramatically differed between QA-treated and -untreated tissues at three temperatures (Fig. 5A). To eliminate the effect of the differential total levels of *frq* mRNA between the QA-treated and -untreated tissues, the amount of each spliced species was normalized to the total amount, and the statistics indicate that the ratios of variants V and VII significantly increased, while the ratio of variant III decreased (Fig. 5B). These results suggest that the exosome plays differential roles in the regulation of these variants by differentially regulating the decay, splicing, or a combination thereof.

In the samples treated with thiolutin for 0 and 15 min, the decay rates of variants III, VI and VII, but not that of variant IV, were significantly slower in the ds*rrp44* strain treated with QA (Fig. 5C). These results suggest that the degradation of *frq* variants are differentially regulated, and the degradation of variants III, VI and VII might be under the control of the exosome, while variant IV might be controlled by alternative mRNA decay pathways. Moreover, because variant III primarily determines the level and ratio of I-6-containing *frq* transcripts, an increase in the stability of variant III upon *rrp44* knockdown suggests an increase in the ratio of I-6-containing *frq* transcripts. However, the level of variant III in the presence of QA was less than that in the absence of QA (Fig. 5A,B), suggesting that the exosome participates in the regulation of splicing in addition to the degradation of variant III.

Because the ratio of l-FRQ to s-FRQ is critical for clock function, we next investigated whether the exosome regulates the levels of l-FRQ/s-FRQ by affecting *frq* splicing. We measured the levels of both the transcript variants of *frq* mRNA and the protein isoforms, and the strategy is schematically shown in Fig. 5D. RT-PCR with primers flanking *frq* I-6 was performed to detect the levels of mRNAs either excluding or including I-6 in the ds*rrp44* and WT strains at three different temperatures with or without QA addition. The RT-PCR results showed that the level of mRNA excluding I-6 was increased after *rrp44* knockdown (Fig. 5E,F). The alternative splicing of *frq* determines the ratio of the FRQ protein isoforms l-FRQ and s-FRQ. Because FRQ is so extensively phosphorylated that the phosphorylated l-FRQ and s-FRQ proteins cannot be easily distinguished, we conducted FRQ-IP followed by λ PPase treatment to compare the dephosphorylated l-FRQ and s-FRQ. The results showed that the ratio of s-FRQ/l-FRQ was increased in the ds*rrp44* strain in the presence of QA (Fig. 5G,H), which agrees with the RNA analysis. These data demonstrate that the exosome is involved in the regulation of the alternative splicing of the circadian clock gene *frq* and consequently the ratio of FRQ isoforms.

## Discussion

The exosome plays important roles in the regulation of a variety of molecular and physiological processes in different organisms, including mitotic regulation, growth, oxidative stress responses and carcinoma progression^42^,^43^,^44^,^45^. The aberrant activity of the exosome may cause various diseases. For example, the over-expression of Dis3/Rrp44 is associated with adenoma to carcinoma progression in colorectal cancer^42^, and the over-expression of Rrp46 contributes to chronic myelogenous leukemia (CML)^46^. At the molecular level, the nuclear exosome regulates the processing of a number of RNA species including rRNAs and sn/snoRNAs, and surveillance and degradation of RNA precursors, including pre-mRNAs, pre-sn/snoRNAs, pre-rRNAs and pre-tRNAs in the nucleus. Whereas, the cytoplasmic exosome regulates the degradation of mRNAs in the cytoplasm^5^,^31^.

The exosome is closely associated with complexes involved in mRNA processing to regulate gene expression at the post-transcriptional level in a coordinated manner. The components of the Ccr4-Not complex directly interact with the exosome, which may serve as a platform for the association between the exosome and TRAMP^47^. In the non-sense mediated mRNA decay (NMD) pathway, the exosome is responsible for the 3′ → 5′degradation of the premature termination codon (PTC)-containing transcripts^48^. The core NMD factor Upf1 physically interacts with the N-terminal domain of Ski7, which mediates the exosome-directed decay of NMD targets^49^. These facts suggest that the post-transcriptional regulation is under the control of a complex network in which the functions of different regulators may be mutually controlled. The exosome also likely cross-talks with the spliceosome. The exosome components Rrp6 and Mtr4 interact with tri-snRNP-associated proteins in HeLa and HEK293 cells^50^. In yeast, more than half of the intron-containing genes are degraded by the exosome, and the poorly spliced pre-mRNAs and the RNAs contain the longer introns are more susceptible to degradation^31^. In addition, the TRAMP complex promotes optimal pre-mRNA splicing by facilitating the recruitment of splicing factors and the rapid degradation of the spliced-out introns by the nuclear exosome^51^. These facts suggest that the exosome may be involved in the regulation of splicing; however, whether and how the exosome regulates alternative splicing remains unclear.

In this work, we first demonstrated that RRP44, the catalytic subunit of the exosome, modulates a variety of cellular processes, including the spliceosome pathway (Table 1). We further showed that the exosome regulates the gene expression of a number of spliceosome components at the RNA level. Among these components, the RNA decay of several genes is controlled by the exosome, suggesting that they are direct targets for the exosome (Fig. 1). More importantly, we showed that the knockdown of *rrp44* resulted in changes in the assembly of the spliceosome U5 and U6 snRNPs (Fig. 3B). The *U5* snRNA contains a 11-nt loop, which functions to align the 5′ and 3′ exons for ligation during the second step of pre-mRNA splicing^52^. The *U6* snRNA pairs with *U4* snRNA to promote the assembly of pre-spliceosome, and it pairs with *U2* to bind with the 5′ss of an intron to facilitates the first step of splicing^1^. These functions suggest that the knockdown of *rrp44* may affect the assembly and functions of spliceosomal components. The analysis of RNA-seq data indicates that RRP44 is required for the regulation of bulk AS events (Fig. 3C).

Previous studies concentrated on the analysis of changes in the IR events caused by the impairment of the exosome, which provided evidence that the exosome plays a role in the degradation of pre-mRNAs^30^,^31^. In this work, the increase in the ratio of spliced forms of the 179 IR events (Fig. 3C, supplemental Table 1) suggests that the exosome may play additional roles in the regulation of splicing events. An analysis of the AS events of NCU05290, NCU07421 and *frq* (Fig. 3, 4, 5) provided evidence that the exosome may directly influence splicing via the spliceosome. The detailed mechanisms by which the exosome selectively regulates splicing and degradation remain to be further investigated. In all the tested genes, we only identified a few whose splicing is controlled by exosome, and this specificity might be attributed to the SR proteins which are also affected by exosome.

The exosome has been known to regulate the circadian clock in apost-transcriptional fashion by facilitating the decay of *frq* transcripts^25^. Accumulating evidence has demonstrated that alternative splicing is involved in the regulation of the circadian clock^13^,^14^,^18^. Here, we demonstrate that the exosome is involved in the regulation of the alternative splicing of the circadian clock gene *frq* and consequently the ratio of FRQ isoforms. Of the FRQ isoforms, l-FRQ supports a shorter period, whereas s-FRQ supports a longer period^22^,^24^. In addition to the stabilization of *frq* transcripts, the increased splicing of *frq* I-6 may explain the longer circadian period of the ds*rrp44* strain. These data also support that the exosome may exert its function by mediating alternative splicing.

**Figure 6:**
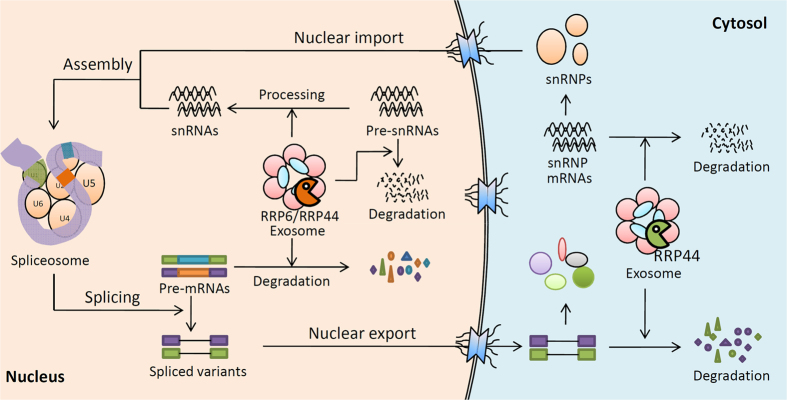
Exosome regulates splicing through multiple pathways. Exosome regulates the levels of unspliced/spliced transcripts in spliceosome-dependent and -independent pathways. In the spliceosome-dependent pathways, the exosome regulates the splicing by affecting the gene expression and assembly of spliceosome and the processing and turnover of snRNAs. In the spliceosome-independent pathways, the exosome mediates the degradation of pre-mRNAs in the nucleus while being responsible for the turnover of both the unspliced and spliced transcripts in the cytoplasm.

In summary, we showed that in addition to the regulation of pre-mRNA degradation, the exosome regulates gene expression and consequently the assembly of the spliceosome complex. Thus, the exosome may regulate the levels of spliced species via spliceosome-dependent and -independent pathways. In the spliceosome-dependent pathways, the exosome regulates the gene expression and assembly of spliceosome and the processing and turnover of snRNAs, which further modulates the function of spliceosome. In the spliceosome-independent pathways, the exosome regulates the degradation of pre-mRNAs in the nucleus and the turnover of both the unspliced and spliced transcripts in the cytoplasm (Fig. 6). These pathways also most likely synergistically regulate the levels of spliced transcripts. Nuclear and cytoplasmic forms of exosome complex might function differently in these processes, the former regulates the processing and degradation of snRNAs and the surveillance of pre-mRNAs, while the latter mediates the mRNA degradation of a set of snRNP and SR protein related genes (Fig. 6).

## Methods

### Strains and growth conditions

The *301-5* (*bd, a*) strain was used as the wild-type (WT) strain. The *301-6-6* strain (*bd, his-3, A*) was used as the host strain for *his-3*-targeting constructs. Liquid cultures were grown in minimal a media (1 × Vogel’s, 2% glucose). When quinic acid (QA) was used, the liquid cultures were grown in media with 0.01 M QA (pH 5.8), 1 × Vogel’s, 0.1% glucose, and 0.17% arginine. The race tube media contained 1 × Vogel’s, 0.1% glucose (0% when QA was used), 0.17% arginine, 50 ng/mL biotin, and 1.5% agar.

### Generation of knockdown strains

The knockdown strains ds*prp5*, ds*U4-2* and ds*prmt5* were generated via the introduction of plasmids that expressed RNA hairpins with complementarity to the gene to be inhibited into the *301-6-6* strain^26^. The hairpin sequence was complementary to ~500 bp of the gene of interest downstream of the *qa-2* promoter. The resulting plasmids were targeted to the *his-3* locus via transformation into *301-6-6* (*bd, his-3, A*). The ds*rrp44* strain was generated as described previously and allows the expression of *rrp44* to be suppressed by the addition of QA^25^.

### RNA and protein analyses

RNA extraction and northern blot analyses were conducted as described previously^53^. Equal amounts of total RNA (10 μg) were loaded onto agarose gels for electrophoresis; the gels were subsequently blotted and probed with RNA probes specific to the genes of interest. RNA degradation was detected by the addition of thiolutin (final concentration 10 μg/ml), which is an inhibitor of RNA polymerase II. The samples were harvested 15 min, 30 min, and 45 min after the addition of thiolutin, and the total RNA was extracted for subsequent RT-PCR detection^25^. For qRT-PCR, the samples were treated with RNase-Free DNase I (NEB) and then subjected to reverse transcription using M-MLV (Invitrogen) and random primers. The synthesized cDNAs were amplified with SYBR Green Master Mix (Takara) using a LightCycler 480 (Roche). The protein extraction, western blot analysis, and immunoprecipitation assays were performed as previously described^54^. Equal amounts of total protein (40 μg) were loaded onto gels (7.5% gels containing a ratio of 37.5:1 acrylamide/bisacrylamide) for SDS-PAGE. The FRQ protein was de-phosphorylated via treatment with Lamda phosphatase (λ PPase, NEB)^54^.

### Generation and validation of PRP5 antibody

The entire ORF of the *Neurospora prp5* gene was cloned into pET-28a (+) and expressed in the BL21 (DE3) *Escherichia coli* strain. Purified His-PRP5 protein was used to immunize rabbits. The obtained antibodies were validated by western blot analysis with the ds*prp5* strain.

### Sucrose fractionation analysis

Sucrose density gradients (10–30%) were prepared, and 4 mg total protein samples were loaded for each analysis. The gradients were centrifuged at 175,000 × g for 18 h in a SW-40 Rotor (Beckman) at 4 °C. Twelve equal fractions were collected, and 450 μl of each fraction was subjected to RNA analysis. The samples were treated with DNase I prior to RT-PCR to determine the levels of *U5* and *U6-2*. Western blot analyses were utilized to determine the distribution of PRP5^38^.

### RNA-seq and analysis

The RNA samples from ds*rrp44* strains grown with or without QA were pooled and processed to prepare the mRNA-seq library using the standard Illumina protocol. The sequencing was performed on an Illumina HiSeq™ 2000 at BGI, China. Tophat was used as an aligner to map the reads to the reference genome (*N. crassa* OR74A (NC12))^55^. Cufflinks was used to reconstruct the transcripts and estimate the gene expression levels^55^. The SplicingViewer tool was used to annotate alternative splicing events^56^. Mixture of Isoforms (MISO) was used to estimate and compare the AS event frequencies between samples^57^. The Bayes factors were calculated by MISO as the presentation of evidence against no difference between the samples^58^. The sashimi plots were graphed for visualization of the analyses of isoform expression with the build-in package sashimi plot of MISO^57^,^59^ (Supplemental information).

### Statistical Analyses

Statistical significance was calculated using Student’s *t*-test. The significance values are *p < 0.05, **p < 0.01 and ^#^*p* < 0.001. n ≥ 3.

## Additional Information

**How to cite this article**: Zhang, L. *et al*. The exosome controls alternative splicing by mediating the gene expression and assembly of the spliceosome complex. *Sci. Rep*. **5**, 13403; doi: 10.1038/srep13403 (2015).

## Supplementary Material

Supplementary Information

Supplementary Table S1

## Figures and Tables

**Table 1:** KEGG pathway analysis of differentially expressed genes (DEGs) in ds*rrp44* strain (QA+ vs. QA−).

Pathway	DEGs with pathway annotation (2384 in total)	*P*-value	Benjamini and Hochberg correction
Pyrimidine metabolism	23 (0.96%)	5.8E-5	4.2E-3
RNA polymerase	13 (0.5%)	3.3E-4	1.2E-2
Spliceosome	28 (1.2%)	7.5E-4	1.8E-2
Purine metabolism	23 (1.0%)	2.1E-3	3.7E-2
Base excision repair	10 (0.4%)	3.1E-3	4.5E-2
DNA replication	13 (0.5%)	5.2E-3	6.1E-2
RNA degradation	15 (0.6%)	1.5E-2	1.4E-1
Basal transcription factors	8 (0.3%)	3.2E-2	2.5E-1

Note: Enrichment of pathways was analyzed by DAVID; *P*-values were adjusted using multiple hypothesis testing and the Benjamini and Hochberg correction^60^.

**Table 2:** snRNP and spliceosome-associated genes up-regulated in ds*rrp44* strain (QA+ vs. QA−).

Gene ID	Gene name	Fold change	*P*-value	Functional classification
NCU07548	*snp1*	2.265586	3.09E-75	U1 related
NCU10810	*prp39*	2.715431	0	U1 related
NCU06959	*snu71*	2.140219	4.1E-87	U1 related
NCU05645	*luc7*	3.219791	3.36E-55	U1 related
NCU03062	*prp40*	2.861535	1.9E-262	U1 related
NCU02696	*prp5*	2.223039	1.8E-169	U1 related
NCU02572	*snu114*	2.068642	6.61E-86	U5 related
NCU02685	*brr2*	2.156961	4E-307	U5 related
NCU07832	*prp8*	2.185044	0	U5 related
NCU07265	*snu23*	3.634172	1.71E-26	U5 related
NCU11222	*snu66*	2.632671	1.3E-154	U4/6 related
NCU01637	*prp3*	2.538265	4.39E-78	U4/6 related
NCU01331	*snu13*	3.047531	2.7E-178	U4/6 related
NCU08036	*syf1*	2.183291	7.7E-135	PRP19 complex
NCU00664	*isy1*	2.748446	2.73E-55	PRP19 complex
NCU02966	*prp46*	2.143234	9.91E-51	PRP19 complex
NCU01613	*prmt5*	1.6094078	0	Spliceosome-associated

Note: The fold changes of expression between QA+ and QA− were calculated from RNA-seq data.
