# Supplementary material for: The exosome controls alternative splicing by mediating the gene expression and assembly of the spliceosome complex
Source: Sci Rep. 2015 Aug 26;5:13403. doi: 10.1038/srep13403 (PMC4549623; doi:10.1038/srep13403)
Supplement: Supplementary Information [file srep13403-s1.doc]

# The exosome controls alternative splicing by mediating the gene expression and assembly of the spliceosome complex

Lin Zhang ^a^, Yufeng Wan ^a,†^, Guobin Huang ^a^, Dongni Wang ^a^,

Xinyang Yu ^a,‡^, Guocun Huang ^b^, Jinhu Guo ^a, *^

^a^ State Key Laboratory of Biocontrol, Key Laboratory of Gene Engineering of the Ministry of Education, School of Life Sciences, Sun Yat-sen University, Guangzhou 510006, China.

^b^ Centre for Circadian Clocks, Medical College, Soochow University, Suzhou 215123, China.

^†^ Present address: Department of Biology, Texas A&M University, College Station, Texas 77843, USA.

^‡^ Present address: School of Biomedical Sciences, University at Buffalo, Buffalo, New York 14260, USA.

Corresponding author: Jinhu Guo

School of Life Sciences, Sun Yat-sen University

132 E Waihuan Rd, High Education Mega Center

Guangzhou 510006 China

Phone/Fax: 86-20-39332939

E-mail: [guojinhu@mail.sysu.edu.cn](mailto:guojinhu@mail.sysu.edu.cn)

Supplementary information:

**Results**

Figure 1

**
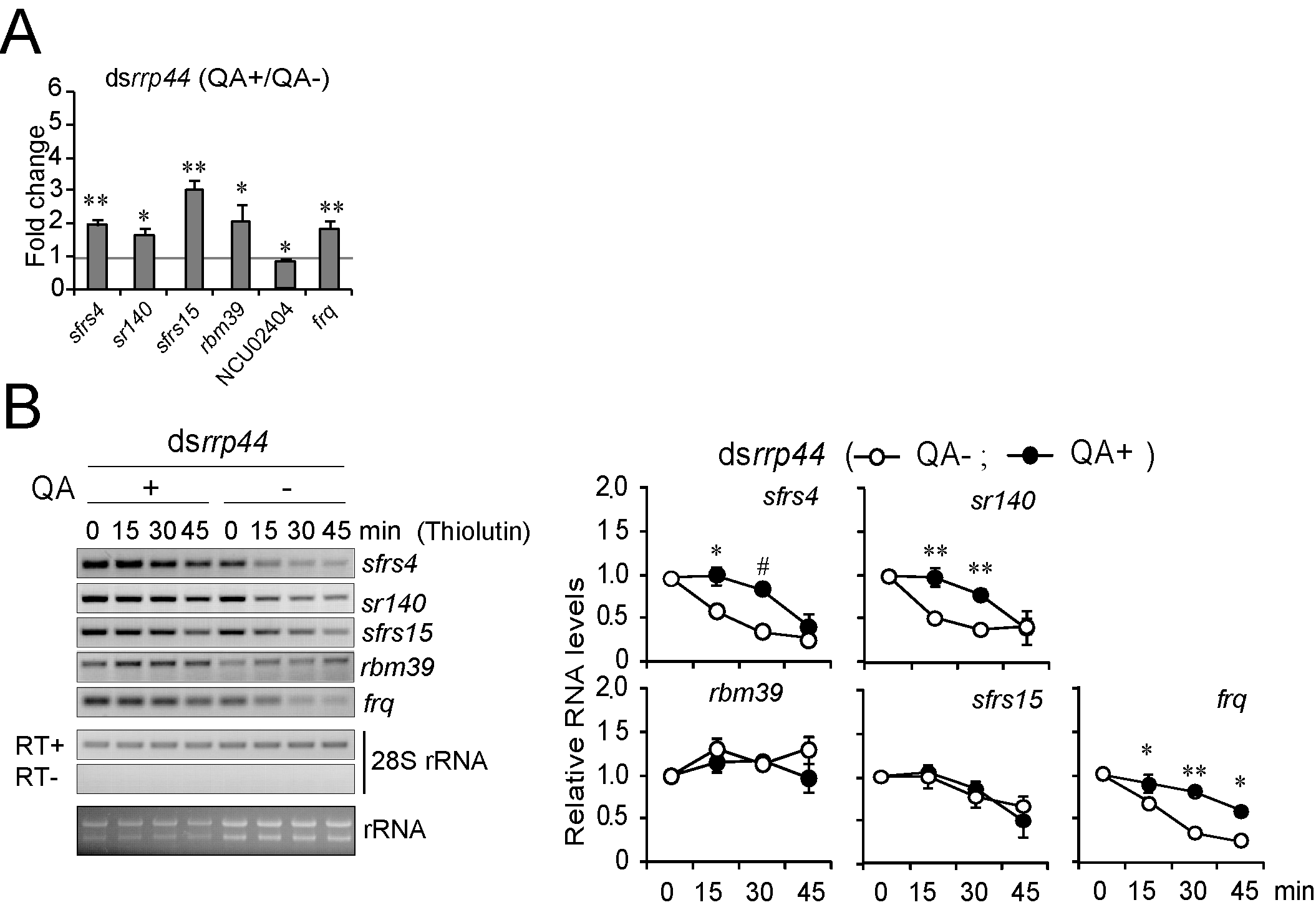
**

Fig. 1 Expression of four SR protein related genes in the ds*rrp44* strain. (A) qRT-PCR analyses of the expression of four SR protein related genes in the ds*rrp44* strain, QA+ versus QA-. NCU02404 and *frq* served as control showing reduction and induction of levels, respectively. (B) RT-PCR of four mRNAs encoding SR or SR related proteins. Thiolutin was added to block the transcription. Samples were harvested at 0, 15, 30, and 45 min after thiolutin addition. Left panel: Representative results of three independent experiments. Right panel: Densitometric analysis of the mRNA decay results, the RNA levels at 0 min was set to 1. The results of *frq* served as control. Data represent means ± SEM of three independent experiments, * *p*< 0.05, ** *p*< 0.01, # *p*< 0.001, QA+ versus QA-.

**Methods**

Analysis of RNA-seq and splicing

RNA samples from independent triplicates were pooled for mRNA-seq library construction by following Illumina standard protocol. The sequencing was performed by Illumina HiSeq™ 2000 (BGI, China). Tophat software was used to map the reads to the reference genome (*[N. crassa](http://www.broadinstitute.org/annotation/genome/neurospora/GenomeDescriptions.html#NC12)* OR74A (NC12)) ^1^. Cufflinks was used to reconstruct the transcripts and estimate the value of gene expression ^2^. SplicingViewer was used to annotate the alternative splicing events ^3^. Mixture of Isoforms (MISO) was used to estimate and compare the AS events frequency between different samples ^4^. The Bayes factors were calculated by MISO as the presentation of evidence against no difference between the samples.

Detailed Calculation steps and parameters

1. Map the RNA-seq reads to the reference genome (*[N. crassa](http://www.broadinstitute.org/annotation/genome/neurospora/GenomeDescriptions.html#NC12)* OR74A (NC12)) with Tophat.

Parameters: allowed mismatches=3

Minimum intron size=15

Maximum intron size=5000

Pair-ended mapping

1. Annotate the AS events with SplicingViewer [Liu et al 2011] by following the standard protocol described on its website (http://bioinformatics.zj.cn/splicingviewer/document.php).
2. Group and format the AS annotation for MISO analysis, and the results retrieve four types of splicing events: 5’ss, 3’ss, exon skipping (ES) and intron retention (IR).
3. Estimate and compare the frequency of each alternative splicing event (psi, percent spliced in, Ψ) by (Mixture of Isoforms) MISO with default settings ^4^. The data were filtered by following parameters:

Number of total reads aligning to any isoform≥10.

The absolute Δ Ψ value≥0.10.

The bayes-factor≥10.

1. Perform statistical tests to compare the significance between the expression of different splicing variants. Bayes factors estimated by MISO were used for the determination. Bayes factor can be interpreted as the weight of evidence in data *D*, which is in support of *H_1_* over *H_0_* ^4^:


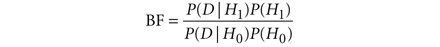


In our case, BF≥10 means that in a certain alternative splicing event, there is no strong evidence supporting a difference between two samples ^5^.

**References**

1. Trapnell, C., *et al*. Transcript assembly and quantification by RNA-Seq reveals unannotated transcripts and isoform switching during cell differentiation. Nature Biotechnology. *28*, 511-515 (2010).
2. Trapnell, C., Pachter, L., and Salzberg, SL. TopHat: discovering splice junctions with RNA-seq. Bioinformatics. *25*, 1105-1111 (2009).
3. Liu, Q., Chen, C., Shen, E., Zhao, F., Sun, Z. & Wu, J. Detection, annotation and visualization of alternative splicing from RNA-Seq data with SplicingViewer. Genomics. *99*, 178-182 (2012).
4. Katz, Y., Wang, E.T., Airoldi, E.M.& Burge, C.B. Analysis and design of RNA sequencing experiments for identifying isoform regulation. Nature Methods. *7*, 1005-1015 (2010).
5. Kass, R.E. & Raftery, A.E. Bayes factors. J. Am. Stat. Assoc. *90*, 773-795 (1995).
